# Incretin and glucagon receptor polypharmacology in chronic kidney disease

**DOI:** 10.1152/ajpendo.00374.2023

**Published:** 2024-03-13

**Authors:** Brandon E. McFarlin, Kevin L. Duffin, Anish Konkar

**Affiliations:** Lilly Research Laboratories, Lilly Corporate Center Indianapolis Indiana United States

**Keywords:** chronic kidney disease, diabetes, GLP-1, incretins, obesity

## Abstract

Chronic kidney disease is a debilitating condition associated with significant morbidity and mortality. In recent years, the kidney effects of incretin-based therapies, particularly glucagon-like peptide-1 receptor agonists (GLP-1RAs), have garnered substantial interest in the management of type 2 diabetes and obesity. This review delves into the intricate interactions between the kidney, GLP-1RAs, and glucagon, shedding light on their mechanisms of action and potential kidney benefits. Both GLP-1 and glucagon, known for their opposing roles in regulating glucose homeostasis, improve systemic risk factors affecting the kidney, including adiposity, inflammation, oxidative stress, and endothelial function. Additionally, these hormones and their pharmaceutical mimetics may have a direct impact on the kidney. Clinical studies have provided evidence that incretins, including those incorporating glucagon receptor agonism, are likely to exhibit improved kidney outcomes. Although further research is necessary, receptor polypharmacology holds promise for preserving kidney function through eliciting vasodilatory effects, influencing volume and electrolyte handling, and improving systemic risk factors.

## INTRODUCTION

Chronic kidney disease (CKD) is a global health concern characterized by the progressive loss of kidney function, measured as a reduction in estimated glomerular filtration rate (eGFR). Despite substantial investments into understanding the (patho)physiology of CKD, standard-of-care therapies targeting the renin-angiotensin-aldosterone system (RAAS), including angiotensin-converting enzyme inhibitors (ACEi) and angiotensin receptor blockers (ARBs), have remained unchanged for over two decades (1). However, novel therapies, including sodium-glucose cotransporter 2 inhibitors (SGLT2i) and mineralocorticoid receptor antagonists, are expanding treatment options for patients with CKD (2–4). Nevertheless, there is a significant residual risk for CKD progression, and overall mortality remains high (5–8). In addition to the rise in leading risk factors of CKD, namely hypertension and type 2 diabetes (T2D), obesity has recently been recognized as an independent risk factor for CKD (9–11). The link between these leading risk factors and CKD is multifaceted, as hyperglycemia, hypertension, insulin resistance, and inflammation intertwine to exacerbate kidney damage and compromise kidney function (12).

In recent years, the use of incretin-based therapies, particularly glucagon-like peptide-1 receptor agonists (GLP-1RAs), have gained traction as novel glucose- and body weight-lowering agents (13–15). Incretin analogs possessing dual and triple pharmacological activities have yielded greater improvements in glycated hemoglobin levels (HbA_1C_, a marker of long-term glucose control) and weight management in patients with T2D and obesity (16–20). Additionally, emerging evidence, both preclinically and clinically, suggests that these therapies reduce albuminuria, reduce blood pressure, and may preserve kidney function (21–24). This review aims to explore the evidence concerning incretin receptor mono- and polypharmacology as a viable therapeutic approach for patients with CKD and offer a framework for how these therapies impact the kidney.

## BRIEF HISTORY OF GLUCAGON AND INCRETIN HORMONES

In 1922, Kimball and Murlin (25) identified glucagon as a potent antihypoglycemic factor released by the pancreas, responsible for increasing blood glucose levels in the face of hypoglycemia. Glucagon, a 29 amino acid hormone, is produced by pancreatic α-cells and acts through the glucagon receptor (GCGR) in various tissues (26–29). During the 1970s to 1990s, glucagon research surged, and its biological actions were studied in various metabolic processes, including central regulation of food intake, adipose thermogenesis, inhibition of gastrointestinal motility, modulation of lipid metabolism, cardiac output, and autophagy (30). It was during this period that researchers also began to understand the impact of glucagon on kidney function, including its influence on glomerular filtration and water reabsorption (31, 32). Despite these findings, over a century after its discovery, glucagon’s clinical use has primarily remained as an emergency treatment for severe hypoglycemia.

In 1929, Zunz and La Barre (33, 34) identified gut extracts that potentiated glucose-dependent insulin secretion and coined the term “incretin.” The key incretins were identified as glucose-dependent insulinotropic polypeptide (GIP) in 1973 and glucagon-like peptide 1 (GLP-1) in 1983 (35–41). GIP, a 42 amino acid hormone, is secreted from enteroendocrine K cells located primarily in the duodenum and jejunum, while GLP-1 is a 30–31 amino acid hormone primarily produced by enteroendocrine L cells in the ileum and colon by posttranslational processing of proglucagon (42, 43). Together these hormones play essential roles in regulating glucose homeostasis, insulin and glucagon secretion, and metabolism (15, 44, 45). Synthetic analogs that mimic the actions of these endogenous hormones are receiving significant attention as novel antidiabetic and antiobesity therapies. Furthermore, early clinical evidence suggests that these hormone mimetics may also show benefits in the treatment of patients with CKD.

## INCRETIN ACTIONS IN THE KIDNEY

Approval of the first GLP-1RA, exenatide, by the FDA nearly two decades ago, catalyzed a succession of similar agents for the treatment of T2D and later obesity. Most data from cardiovascular outcome trials (CVOT) demonstrate that GLP-1RA treatment translates to improved kidney function, either directly or indirectly (46). Systematic reviews and meta-analysis of trial results in patients with T2D treated with GLP-1RAs reveal a modest reduction in composite kidney outcomes, defined as the development of new-onset macroalbuminuria, decline in eGFR (or increase in creatinine), progression to end-stage kidney disease (ESKD), or death attributable to kidney causes (47). Below, we discuss possible physiological mechanisms, clinical evidence, and outline areas necessitating further investigation to better understand the potential application of GLP-1RAs for the treatment of CKD. GIP receptor agonists also show promising antidiabetic and antiobesogenic effects as monotherapy and are currently in clinical development (48, 49). However, GIP will not be a major focus of this review due to the lack of concrete evidence for GIP receptor (GIPR) expression or impact on the kidney. Instead, readers may consider recent reviews on the topic (14, 50).

### Effects of GLP-1 on the Kidney

**Figure 1.**
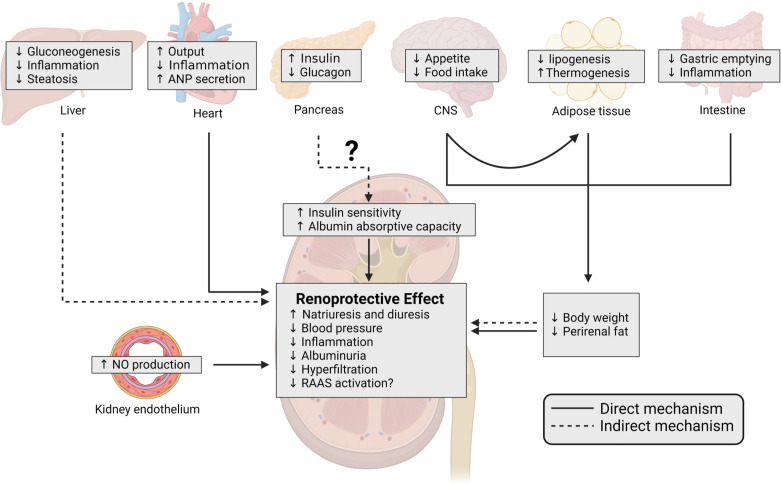
Schematic of direct and indirect effects of glucagon-like peptide-1 receptor (GLP-1R) activation by GLP-1 and analogs on the kidney. GLP-1 and GLP-1RAs exert a myriad of downstream impacts on peripheral tissues, capable of influencing the kidney either directly or indirectly. GLP-1R activation indirectly enhances insulin sensitization in the liver by stimulating PDK-1, Akt, protein kinase C, and peroxisome proliferator-activated receptor-γ, which collectively contribute to reduced steatosis (55, 56). This effect is further complemented by the inhibition of natural killer T cells and the promotion of anti-inflammatory gene expression, coupled with the suppression of profibrotic gene expression (57–59). In the pancreas, GLP-1 released from pancreatic α-cells activates GLP-1R to induce insulin secretion and diminishes glucagon release (60). Notably, podocytes in the kidney express proteins involved in insulin signaling, and hyperglycemia is a major contributor to podocyte damage. However, despite the absence of GLP-1R in podocytes, GLP-1 analogs improve glomerular insulin sensitivity, reduce glomerular albumin permeability, and improve albumin uptake in proximal tubule (61–64). Collectively, these effects work in concert to lower albuminuria. Within the heart, GLP-1R activation can augment blood flow and output. This activation also stimulates atrial natriuretic peptide (ANP) secretion, which promotes lower blood pressure by increasing natriuresis and diuresis and contributes to anti-inflammatory effects that likely contribute to reductions in atherosclerosis (65). In the central nervous system (CNS), GLP-1R activation plays a key role in modulating energy homeostasis by eliciting appetite suppression, reduced food intake, enhanced thermogenesis, and reduced lipogenesis in adipose tissue (14, 15, 66, 67). In the gastrointestinal tract, GLP-1R signaling mitigates dysbiosis, inflammation, and slows gastrointestinal transit (68–72). Cumulatively, the impact on CNS, adipose tissue, and the gastrointestinal tract culminate in diminished body weight and adiposity, both of which directly and indirectly impact kidney outcomes. For example, reductions in excessive visceral adiposity can alleviate kidney compression, leading to improved electrolyte handling and blood pressure regulation (11, 73). Concurrently, body weight reduction improves hemodynamics, blood pressure, and the microbiota, all of which indirectly contribute to improved kidney outcomes (11). The kidney endothelium expresses GLP-1Rs, which directly influence systemic and kidney vasculature, promoting the release of nitric oxide (NO), which results in vasodilation and reduces blood pressure (74). While GLP-1R presence along the nephron lacks definitive evidence, GLP-1 and analogs indirectly reduce proximal tubule sodium/hydrogen exchanger isoform 3 (75, 76). This effect is further reinforced by diminished renin-angiotensin-aldosterone system (RAAS) activation, amplifying natriuresis and diuresis (77–80). Additionally, vasodilation of the afferent arteriole and luminal natriuresis could stimulate tubuloglomerular feedback, countering increased luminal flow and ultimately reducing estimated glomerular filtration rate decline (81, 82). In the diagram, solid lines denote direct mechanisms, while dashed lines indicate indirect or secondary mechanisms through which GLP-1R activation could enhance kidney function. Figure was created with BioRender.com.

Although the influence of GLP-1RAs on kidney hemodynamics is unclear, some studies have shown that GLP-1RA treatment can acutely elicit vasodilation of the afferent arterioles (51–53). However, these findings are contradicted by observations in patients with T2D in which acute GLP-1RA treatment increases vascular resistance in the afferent arteriole, without an impact on the efferent arteriole (54). Administration of native GLP-1 and GLP-1RAs in preclinical rodent models elicits increases in natriuresis, diuresis, renal blood flow (RBF), and GFR (Fig. 1) (51, 74, 81, 83–87). Comparable responses of increased natriuresis, diuresis, RBF, and GFR were also observed clinically following short-term GLP-1 or GLP-1RA administration, albeit with notable variability (51, 52, 77, 83–85, 88). Additionally, micropuncture studies show GLP-1RAs increase single nephron GFR by 33–50% (82). These potential vasodilatory effects are likely mediated by increased nitric oxide (NO) production and availability (52, 81). Collectively, these observations suggest the presence of functionally active GLP-1Rs in the kidney.

Despite intensive exploration, discordant findings of GLP-1R expression in various tissue and cell types are likely due to the lack of validated tools with high specificity to the receptor (51, 84, 89–93). Studies utilizing validated monoclonal antibodies described the expression of GLP-1Rs in smooth muscle cells in the wall of arterioles and arteries in primates (94). These GLP-1R-expressing cells were associated with juxtaglomerular cells and exhibited a degree of overlap with renin-secreting cells. Importantly, a similar expression profile was observed in the human kidney (94). It is well-established that reducing blood pressure is kidney protective and the RAAS plays a key role in blood pressure regulation as demonstrated in clinical trials and subsequent approval of ACEi and ARBs (95). Angiotensin II (ANG II) can exert its effects in both the vasculature and the kidney by binding to angiotensin type 1 receptors to stimulate vasoconstriction and fluid reabsorption in the kidney and increase blood pressure. While the influence of GLP-1RA on blood pressure regulation is inconsistent and poorly understood, there is evidence that GLP-1RA can modestly lower systolic blood pressure (17, 96–98). Studies in healthy volunteers and patients with T2D showed that acute administration of GLP-1RAs can lower circulating ANG II levels (77–79, 99). Whether lower circulating ANG II is a function of lower production of RAAS upstream components (i.e., angiotensinogen, renin, or ACE1) or enhanced breakdown into the counterregulatory RAAS peptide ANG II(1–7) is poorly understood. The aforementioned studies also showed no change in or lowering of plasma renin levels with acute GLP-1RA treatment (77–79, 99). Consistent with reduced RAAS activity, recent clinical trials showed that long-term treatment with some GLP-1RAs modestly reduced systolic blood pressure, although notable variability has been observed (75, 100). Since GLP-1R expression appears confined to the vasculature within the kidney, it is likely that any physiological and pharmacological impacts associated with GLP-1 and GLP-1RAs on kidney function, apart from their direct vasculature effects, are indirect and stem from GLP-1R activity in peripheral tissues and central nervous system (Fig. 1).

Despite the lack of evidence for the presence of GLP-1Rs along the nephron, GLP-1 and GLP-1RAs have also been implicated in volume and electrolyte homeostasis. Acute GLP-1RA administration increases sodium excretion in both rodents and humans (52, 54, 78, 101). The sodium/hydrogen exchanger isoform 3 (NHE3), which is pivotal to fluid and sodium reabsorption in the proximal tubule and acid/base homeostasis, appears to be involved in GLP-1R-induced natriuresis and diuresis (84, 85, 102). Administration of GLP-1 or GLP-1RAs in rodent models leads to phosphorylation of NHE3 at the protein kinase A consensus sites Ser552 and Ser605 that causes inhibition of the antiporter (85, 92, 101, 103, 104). In support of the findings implicating the GLP-1-GLP-1R-NHE3 axis, several studies show that acute GLP1-RA treatment increases urinary pH and lithium clearance, the latter a marker of volume flow from the proximal tubule (77, 78, 88). It is also possible that GLP-1 and GLP-1RAs drive natriuresis indirectly through atrial natriuretic peptide (ANP). Preclinically, GLP-1RA treatment increases circulating ANP levels, which would contribute to natriuresis (105). However, this effect has not been definitively demonstrated in humans (106, 107). Administration of GLP-1RAs in patients with T2D reveals that GLP-1RA-stimulated natriuresis is preserved in this population at early time points following treatment initiation (108, 109). However, chronic GLP-1RA treatment, with long-acting GLP-1RAs, shows no change in electrolyte handling over time, which suggests either desensitization of GLP-1Rs or the presence of adaptive mechanisms to counter the effects seen acutely or at early treatment time points (110). In addition to natriuresis, preclinical evidence suggests that GLP-1RAs may play a role in potassium handling. Acute GLP-1RA treatment in Wistar rats exhibited marginal increases in potassium excretion under basal conditions, whereas potassium excretion increased 2-fold within 1 hour of treatment in conditions of hyperkalemia (111). However, this finding has not been corroborated in studies in humans.

### Impact of GLP-1 on Risk Factors Affecting the Kidney

Hyperglycemia is a key risk factor contributing to CKD development and progression, and improved glycemic control contributes to delayed kidney disease progression. The United Kingdom Prospect Diabetes study (UKPDS) and The Actions in Diabetes and Vascular Disease: Preterax and Diamicron MR Controlled Evaluation (ADVANCE) trials demonstrated that intensive glucose control alone reduced the risk of albuminuria by 33% and ESKD by 46%, respectively (11, 73, 112–114). These studies provide evidence of a causal link between dysglycemia and CKD. It is now well established that GLP-1RAs are potent antidiabetic agents that stimulate glucose-dependent insulin secretion and improve glycemic control, as evidenced by reduced HbA_1C_ levels. Given the established role of GLP-1RAs in long-term glycemic control, it is likely that improvement in albuminuria observed with GLP-1RA treatment is, at least partially, mediated by reduced blood glucose levels (Fig. 1) (115, 116). In fact, mediation analysis suggests that more than 50% of the kidney protective effect of GLP-1RAs is attributed to the lowering of HbA_1C_ alone (117, 118). In addition to hyperglycemia, excess adiposity, a major risk factor for hypertension, diabetes, and cardiovascular disease, contributes to an increased risk of CKD (11, 73, 114). Body weight loss is independently associated with a reduction in urinary albumin-to-creatinine ratio (UACR), and previous studies provide evidence for improvements in RBF, inflammatory status, oxidative stress, and kidney hypoxia following body weight reduction (114, 119–121). Readers are directed to recent in-depth reviews on this topic (11, 73). Thus the body weight loss produced by GLP-1RAs in patients with T2D and/or obesity is another indirect action conferring kidney protective benefits (Fig. 1) (19, 122).

GLP-1 and GLP-1RA action have also been shown to improve systemic risk factors that affect kidneys, including inflammation, oxidative stress, and endothelial dysfunction (Fig. 1) (14, 123, 124). GLP-1RA treatment increases NO synthase activity and NO bioavailability thus improving endothelial dysfunction (54, 78, 125–132). The transcription factor nuclear factor-E2-related factor 2 (Nrf2) pathway, which plays an important role in antioxidant actions, has been implicated as a likely mechanism through which GLP-1RAs mediate antisenescent and antioxidative effects (133, 134). Additionally, activation of Nrf2-antioxidant response element pathway attenuates hyperglycemia-mediated podocyte injury in mouse models (133). Additionally, GLP-1 and GLP-1RAs treatment can reduce levels of proinflammatory cytokines, adhesion molecules, and profibrotic signaling that all contribute to improvements in inflammatory, oxidative, and fibrotic status (98, 129, 135–140).

### Clinical Trial Evidence for GLP-1RA Actions on Kidney Outcomes

Currently, no GLP-1RA has received regulatory approval for treating patients with CKD. Among the GLP-1RAs, semaglutide is the only GLP-1 analog being investigated in a dedicated phase 3 clinical trial to assess its impact on kidney outcomes in patients with T2D and CKD (141). However, several phase 3 CVOT studies conducted on GLP-1RAs in participants with T2D, in the presence of cardiovascular disease and other risk factors, have reported kidney outcomes as secondary results (17, 18, 96–98, 136, 137, 142). Below, we provide a concise overview of key findings relevant to kidney outcomes.

In the ELIXA trial, 6,068 participants were randomized to receive either lixisenatide (*n* = 3,034) or placebo (*n* = 3,034) and were followed for a median of 2.1 yr (142). Lixisenatide, a short-acting GLP-1RA, demonstrated a reduction in UACR in participants with T2D and recent coronary event and micro- or macroalbuminuria. However, there was no effect observed on eGFR decline. A post hoc analysis revealed that lixisenatide reduced the progression of UACR in participants with macroalbuminuria, and the effect persisted following adjustments for traditional kidney risk factors and on-trial HbA_1C_ (138). Due to the low occurrence of hard kidney endpoints (e.g., eGFR decline or doubling of serum creatinine), the impact of lixisenatide on these measures could not be definitively established in the studied population (138).

In the REWIND trial, which included 9,901 participants with T2D and previous cardiovascular events or risk factors, participants were assigned to receive either the long-acting GLP-1RA dulaglutide (*n* = 4,949) or placebo (*n* = 4,952) (96). An exploratory analysis of the study revealed that once-weekly administered dulaglutide, for a median period of 5.4 yr, reduced the risk of composite kidney outcomes (defined as new onset macroalbuminuria, a sustained ≥30% decrease in eGFR, or renal replacement therapy) compared with placebo (96). Dulaglutide elicited a reduction in all three components of the composite kidney outcome, with the most notable effect seen in the development of macroalbuminuria. AWARD-7, a study involving participants with T2D and moderate-to-severe CKD (*n* = 577), investigated the effect of once weekly dulaglutide (0.75 and 1.5 mg) treatment compared to insulin glargine for 1 yr (139). Study results indicated that dulaglutide effectively slowed the decline in eGFR and reduced UACR in participants with baseline macroalbuminuria compared to insulin glargine. A post hoc analysis showed that dulaglutide (1.5 mg) reduced the risk of the composite outcome of ≥40% eGFR decline or ESKD in participants with or without baseline macroalbuminuria (140).

In the LEADER trial, 9,340 participants with T2D and high cardiovascular risk were randomized to receive liraglutide (*n* = 4,668) or placebo (*n* = 4,672) and were followed for a median period of 3.8 yr (18). A prespecified analysis of the secondary kidney outcomes, involving a composite of new-onset macroalbuminuria, doubling of the serum creatinine level, ESKD, or kidney disease-related death, demonstrated that liraglutide significantly reduced the rates of negative kidney outcomes (143). This effect was largely attributed to a decrease in the incidence of macroalbuminuria.

The SUSTAIN-6 trial included 3,297 participants with T2D and high cardiovascular risk who were randomized to receive the long-acting GLP-1RA semaglutide (0.5 or 1 mg; *n* = 1,648) or placebo (*n* = 1,649) for 2 yr (17). Semaglutide elicited a significant reduction in new or worsening nephropathy, defined as new onset macroalbuminuria, doubling of serum creatinine level, reduced creatinine clearance (<45 mL/min/1.73 m^2^), renal replacement therapy, or kidney disease-related death. PIONEER-6 investigated an oral form of semaglutide in 3,183 participants with T2D and high cardiovascular risk, revealing that orally administered semaglutide lowered systolic blood pressure (98). Although no differences in eGFR were observed in PIONEER-6, a post hoc analysis of the results from SUSTAIN-6 and PIONEER-6 trials suggested that semaglutide treatment could have a benefit on eGFR decline (144). In the phase 3 FLOW trial, 3,534 participants with T2D and CKD were randomized to receive semaglutide (1.0 mg) or placebo as an adjunct to standard of care to investigate kidney outcomes for prevention of progression of kidney impairment and risk of kidney and cardiovascular mortality (141). The study was halted early based on an interim analysis that met pre-specified criteria for stopping the trial early for efficacy (145). The complete results are expected to be released by the sponsor during the year 2024.

The AMPLITUDE-O trial involved 4,076 participants with T2D, a history of cardiovascular disease or CKD, and at least one other cardiovascular risk factor. These participants were randomized to receive 4 or 6 mg efpeglenatide (*n* = 2,717) or placebo (*n* = 1,359) for 1.8 yr (97). A secondary analysis of composite kidney outcomes, including new-onset macroalbuminuria, a ≥30% increase in UACR from baseline, sustained ≥40% decrease in eGFR, renal replacement therapy, or sustained eGFR <15 mL/min/1.73 m^2^, demonstrated that efpeglenatide significantly reduced the risk of negative kidney outcomes. A subsequent post hoc analysis showed that the higher dose of efpeglenatide also lowered the risk of composite kidney function outcomes, focusing on eGFR decline, renal replacement therapy, or sustained eGFR <15 mL/min/1.73 m^2^ (146).

Other CVOTs, such as Harmony Outcomes and EXSCEL, reported limited kidney outcomes measurements. In Harmony Outcomes, 9,463 participants were randomized to receive albiglutide (*n* = 4,731) or placebo (*n* = 4,732) and were followed for at least 1.5 yr (137). However, no significant differences in blood pressure or eGFR were observed between groups. The EXSCEL trial investigated the impact of once-weekly exenatide over a median of 3.2 yr in 14,752 participants with T2D, showing only a modest reduction in systolic blood pressure (136).

Collectively, data from these CVOTs suggest that the primary impact of GLP-1RAs is improvement in albuminuria. Notably, the improvement in albuminuria is likely mediated, at least partially, by correction of hyperglycemia and a modest reduction in blood pressure, as discussed earlier. However, robust measures of hard kidney endpoints (e.g., ESKD, doubling of creatinine, renal replacement therapy, and kidney disease-related death) as the primary outcome were lacking in these trials. Given the significant beneficial impact of incretins on glycemic control as well as the reduction of risk factors unrelated to hyperglycemia, including adiposity, inflammation, oxidative stress, and endothelial dysfunction, future studies of incretin-based therapies should incorporate these measurements in dedicated kidney outcomes trials in patients with and without diabetes to enhance our understanding of the utility of these therapies in CKD.

## THE ROLE OF GLUCAGON IN THE KIDNEY

Historically, research on glucagon within the context of diabetes has predominantly focused on its counterregulatory effects on insulin. Due to its ability to rapidly reverse hypoglycemia, significant effort has been expended to develop stable glucagon formulations to treat hypoglycemia as well as a partner to insulin in bihormonal pump therapy (147, 148). In addition, most of the innovation in the glucagon field was focused on developing novel glucagon receptor antagonists for the treatment of T2D, due to the impact of glucagon on hepatic glucose production and hyperglycemia (149–151). Despite promising results in preclinical models of T2D, as well as efficacy observed in early clinical studies, the adverse event profile seen in humans, particularly elevated liver fat deposition and liver enzymes, has limited the clinical utility of glucagon receptor antagonists (152). Thus, as noted above, the therapeutic utility of glucagon has been mainly limited to its use as an emergency treatment for severe hypoglycemia in patients with diabetes. The recent development of dual and triple incretin agonists with glucagon receptor agonist (GCGRA) activity has fueled renewed interest in leveraging glucagon pharmacology in metabolic diseases (153, 154). These novel incretin analogs were designed to exploit the impact of glucagon on reducing appetite, increasing energy expenditure, and lipid oxidation (151, 155–157). Thus combining GCGRA activity with incretin receptor agonist activity holds promise for treating several metabolic diseases, including T2D, obesity, and nonalcoholic steatohepatitis/metabolic dysfunction-associated steatohepatitis. Some analogs have shown promising beneficial impacts on kidney function in early-stage clinical studies, while others are still being investigated (e.g., ClinicalTrials.gov identifier NCT05936151) (158). Whether the GCGRA activity in these multifunctional incretin analogs directly or indirectly contribute to kidney benefits remains an active area of investigation.

Although GCGR expression in the kidney is well established and supports its role in kidney physiology, differentiating between direct and indirect actions is still unclear. Notably, glucagon action in the kidney, which has the highest expression of GCGR after the liver, may play a key role in gluconeogenesis (159, 160). Additionally, GCGR expression is lower in patients with T2D and CKD, and its expression is positively correlated with eGFR (161–163). Genetic studies link human GCGR variants to altered electrolyte handling, which complements findings in mice that kidney-specific GCGR knockdown leads to dysregulation in electrolyte and volume homeostasis (164–166). Kidney-specific GCGR knockdown shows altered nitrogen balance, blood pressure, and increased inflammation and fibrosis (166). Together, these findings highlight the involvement of GCGRs in kidney disease (patho)physiology. Below, we examine evidence for glucagon’s direct and indirect action in the kidney.

### Glucagon Action on Glomerular Filtration Rate

The crucial role played by the kidneys in waste excretion is vital for preventing serious and life-threatening health complications. Studies dating back to the 1920s show high protein intake increases kidney mass (167–172). Additionally, there is a strong correlation between protein intake and GFR (173–178). Concomitant elevation in glucagon following a protein-rich meal or amino acid infusion likely mediates the increases in RBF and GFR (179–183). These alterations facilitate the excretion of amino acid catabolism-derived nitrogenous waste (i.e., urea) (173, 184–187). However, long-term elevation in GFR could further contribute to chronic kidney disease progression in which hyperfiltration contributes to kidney function decline (184, 188–190). These findings provide the foundation for physician recommendations that patients with CKD consume a lower protein diet (191). Nevertheless, the advice to lower protein intake has not been validated in large clinical studies (192). Considering glucagon’s role in stimulating liver gluconeogenesis and ureagenesis, glucagon’s effects on kidney function warrant further investigation.

**Figure 2.**
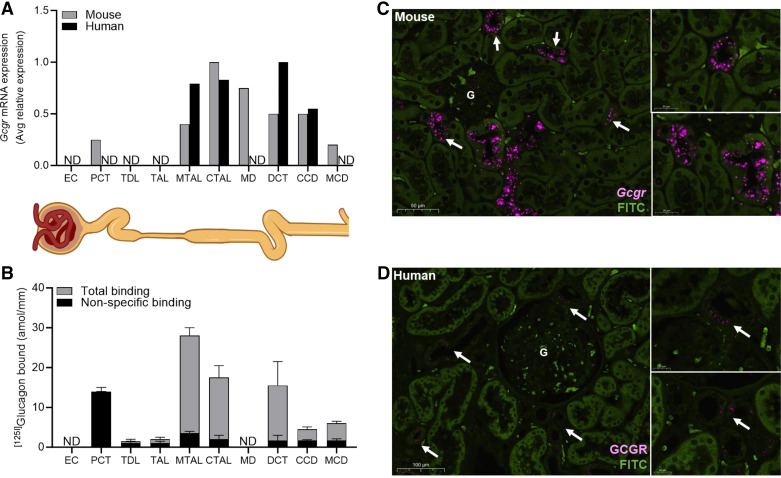
Glucagon receptor expression along the nephron. *A*: relative mRNA expression of glucagon receptor (*Gcgr*) along the nephron of healthy mouse kidney (gray bars) and human kidney tissue (black bars). Mouse relative expression data were adapted from KidneyCellExplorer (cello.shinyapps.io/kidneycellexplorer/; Ref. 200). Human relative expression data are based on data generated by the Kidney Precision Medicine Project accessed on November 2, 2023 (https://www.kpmp.org; Ref. 201), which was funded by the National Institute of Diabetes and Digestive and Kidney Diseases. *B*: distribution of total (gray bars) and nonspecific (black bars) binding of [^125^I]glucagon along the rat nephron adapted from Ref. 202; ND, not determined. *C* and *D*: representative images using RNAscope in situ hybridization of *Gcgr* in mouse kidney tissue (*C*; RNAscope LS 2.5 Probe-Mu-Gcgr; Advanced Cell Diagnostics; cat. no. 417008) or GCGR in human kidney tissue (*D*: RNAscope LS 2.5 Probe-Hu-GCGR; Advanced Cell Diagnostics; cat. no. 406438). Arrows indicate *Gcgr* expression. EC, endothelial cells; G, glomerulus; PCT, proximal convoluted tubule; TDL, thin descending tubule; TAL, thin ascending tubule; mTAL, medullary thick ascending tubule; CTAL, cortical thick ascending tubule; MD, macula densa; DCT, distal convoluted tubule; CCD, cortical collecting duct; MCD, medullary cortical collecting duct.

Hyperglucagonemia can elevate urea synthesis, decrease many circulating amino acids, and increase GFR (193). Interestingly, acute intravenous or portal vein glucagon infusions elicit increases in RBF and GFR, whereas, these alterations were not detected with renal artery infusions (31, 32, 194–197). Notably, only supraphysiological doses of glucagon lead to alterations in GFR (198, 199). Collectively, these observations, along with the lack of evidence for GCGR expression in the glomerulus (Fig. 2), suggest that glucagon’s influence on kidney hemodynamic and filtration are not mediated by direct actions but instead are secondary and likely due to glucagon-stimulated hepatic generation of nitrogenous waste. However, direct actions of glucagon along the nephron could influence GFR through tubuloglomerular feedback, which will be discussed later.

Additional points of consideration, but not directly addressed in this review, are the interplay of glucagon and insulin actions. Insulin, the counterregulatory hormone to glucagon, plays a strong role in modifying glucagon’s systemic impact and more specifically on kidney hemodynamics and GFR. Investigation of glucagon’s role in these parameters must take into account the potential influence on insulin. In fact, correlations between eGFR, glucagon, and insulin show that GFR is more strongly associated with the glucagon-to-insulin ratio compared to either hormone separately (203). The interplay between protein intake, glucagon-to-insulin ratio, and GFR has been recently reviewed elsewhere (184, 204).

### Role of Glucagon along the Nephron

Along the nephron, GCGR is primarily expressed in the thick ascending limb, macula densa, distal tubule, and collecting duct segments (Fig. 2*A*) (161, 200–202, 205). There is also disputed evidence for the presence of GCGR expression in the proximal tubule (202). Gluconeogenesis, a vital but often underappreciated function of the kidney, plays a critical role in kidney and systemic metabolism (206–208). During the fasting state, kidneys account for ∼20–40% of endogenous gluconeogenesis, primarily originating from the proximal tubule (209, 210). Interestingly, gluconeogenic capacity of the kidney might even surpass that of the liver when adjusted for tissue weight (211). However, the influence of glucagon on kidney gluconeogenesis remains unclear. In mice devoid of hepatic glucose release, euglycemia is maintained through compensatory kidney gluconeogenesis and glucagon appears to be involved (212). However, whether glucagon has direct influence on kidney gluconeogenesis or provides gluconeogenic substrates to the kidney is unclear (213–215). Mice with genetic knockdown of GCGR in the kidney show reduced glucose levels during metabolic tolerance tests with various gluconeogenic precursors (i.e., pyruvate and glutamine) and show lower expression of glucose transporters GLUT2 and SGLT2 (166). Additionally, ex vivo studies in rat cortical sections revealed that glucagon can stimulate glucose formation 50–80% above basal levels (216). However, other studies in rat cortical tissue, rat isolated kidneys, and in concious dogs show a lack of effect of glucagon on kidney gluconeogenesis (214, 215, 217). Taken together with the lack of glucagon’s impact on kidney gluconeogenesis in humans (213), it appears glucagon-mediated kidney gluconeogenesis is not supported by conclusive evidence. However, glucagon has been implicated in other actions in the proximal tubule, as discussed below.

At supraphysiological doses, glucagon inhibits electrolyte reabsorption in the proximal tubule (31, 195, 196). Micropuncture studies in hormone-deprived Brattleboro rats, achieved via somatostatin administration, demonstrate that glucagon infusion inhibits water, Na^+^, Cl^−^, K^+^, and Ca^2+^ reabsorption in the proximal tubule (218). Kidney-specific GCGR knockdown in mice increases proximal tubule NHE3 and thick ascending limb Na^+^-K^+^-2Cl^−^ cotransporter 2 expression while lowering epithelial Na^+^ channel subunit expression (166). However, whether the changes in mRNA expression levels correlate with changes in protein levels or their covalent modifications (e.g., phosphorylation) were not explored. Additionally, in vivo micropuncture and microperfusion studies in rats demonstrated that glucagon reduced bicarbonate reabsorption in the proximal tubule, which led to a 45% increase in bicarbonate delivery to the distal tubule (219, 220). These findings implicate glucagon in the maintenance of acid-base homeostasis. Notably, de Rouffignac et al. (218) provide evidence for direct actions of glucagon within the proximal tubule (discussed later). In support of the notion of direct glucagon action in the proximal tubule, Marks et al. (221) reported mRNA expression of *Gcgr* in rat proximal tubules and its influence on facilitating passive glucose transport via increasing glucose transporter proteins. However, radiolabeled glucagon binding studies revealed that the strong glucagon binding observed in the proximal tubule is nonspecific (Fig. 2*B*) (202). Recent transcriptomic analysis conducted in mouse and human kidney tissue show low expression and no evidence of *Gcgr* expression in this segment, respectively (Fig. 2*A*) (161, 200, 201, 222).

Moving further along the kidney nephron, the GCGR is primarily expressed in the distal segments of the kidney (Fig. 2) (161, 200, 202). Glucagon infusion in hormone-deprived Brattleboro rats revealed its ability to stimulate the reabsorption of electrolytes (i.e., Na^+^, Cl^−^, K^+^, and Ca^2+^) and water in the Loop of Henle (218). Increased reabsorption in this region may play an important role in urinary concentrating capacity. In states of elevated circulating nitrogenous waste (i.e., urea), the kidneys can drive greater excretion through its countercurrent multiplication processes (223). Greater reabsorption of solutes (e.g., NaCl) in the thick ascending limb will increase interstitial osmolality and drive greater water reabsorption from the water-permeable inner medullary collecting duct, which will concentrate urea in the collecting duct fluid, where there is low urea permeability, and thus facilitate excretion (223). These effects aid in the excretion of nitrogenous wastes and potentially explain the effect of glucagon-stimulated reabsorption along the Loop of Henle. Relatedly, one intriguing finding is the expression of the GCGR in the macula densa, specialized cells located in the distal tubule segments of the nephron (Fig. 2*A*) (200, 205). These cells, along with the mesangial cells and kidney corpuscle, compose the juxtaglomerular apparatus responsible for detecting changes in tubular fluid composition and transmitting signals to regulate filtration rate and hemodynamics, a process referred to as tubuloglomerular feedback. The possibility of glucagon’s direct interaction with its receptor in the macula densa cells suggests a potential pathway through which glucagon may mediate alterations in hemodynamics and GFR. However, further studies are required to thoroughly investigate this topic. Despite considerable GCGR expression in the distal tubule and collecting duct, less is known about glucagon’s role in these regions. In the distal tubule, glucagon is reported to stimulate Ca^2+^ and Mg^2+^ reabsorption, along with K^+^ secretion, with minimal effects on Na^+^ or Cl^−^ transport (224). Conversely, in the collecting duct, glucagon stimulates water reabsorption and urea excretion by increasing the expression of aquaporin 2 and decreasing urea transporter A1, respectively (225, 226). Together, these studies underscore glucagon’s crucial role in maintaining electrolyte, acid-base, and nitrogen homeostasis.

### Liver-Derived cAMP and Its Role in Kidney Function

Central to the actions of glucagon is its ability to stimulate hepatic gluconeogenesis by inducing intracellular cyclic 3′,5′-adenosine monophosphate (cAMP) production (227–232). Serving as a crucial and ubiquitous secondary messenger, cAMP is generated by adenylyl cyclases and exerts its actions through protein kinase A-mediated pathways. cAMP can also be secreted by various tissues including the liver, adipose tissue, and the kidney, facilitated by organic anion transporters (233–238). Notably, hepatic-derived cAMP can contribute to circulating cAMP content, with implications as both a paracrine or endocrine factor influencing peripheral tissues (239, 240).

**Figure 3.**
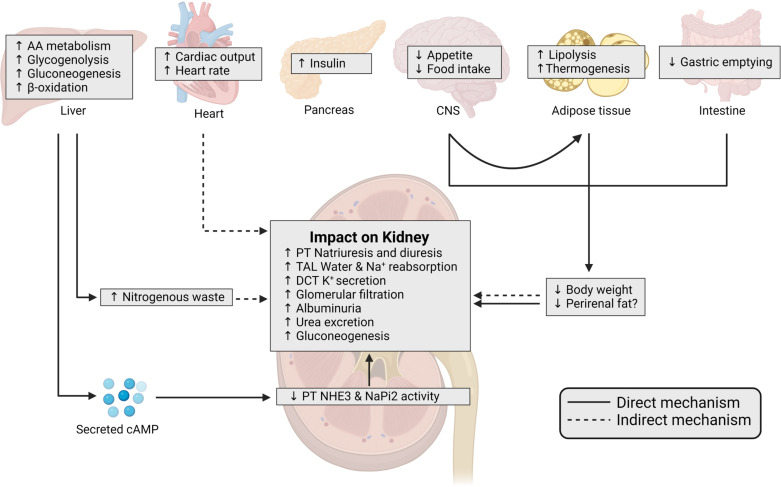
Schematic of direct and indirect effects of glucagon receptor (GCGR) activation by glucagon and analogs on the kidney. GCGR activation can directly and indirectly influence kidney function. GCGR is primarily expressed in the liver and kidneys, with weaker (and controversial) expression observed in adipose tissue, spleen, pancreas, brain, heart, and gastrointestinal tract (28, 243). The primary function of GCGR activation in the liver, where expression is the highest, is stimulation of glycogenolysis and gluconeogenesis to protect against hypoglycemia. Amino acid (AA) metabolism is necessary to drive glucagon-stimulated gluconeogenesis, which consequently leads to increases in nitrogenous waste products. The kidneys are responsible for excreting this waste to prevent its accumulation, which can contribute to increased glomerular filtration rate (GFR) and increased urea excretion along the nephron. Additionally, glucagon-stimulated hepatic cAMP secretion can directly reduce proximal tubule (PT) reabsorption via sodium/hydrogen exchanger isoform 3 (see text). Collectively, the influence of GCGR activation in liver and kidney can contribute to increased GFR, natriuresis, and diuresis. Additionally, hepatic GCGR activation stimulates β-oxidation via carnitine acyl transferase 1-mediated catabolism of fatty acids and inhibition of malonyl-CoA-mediated fatty acid synthesis. Together these effects contribute to lower circulating fatty acid and triglyceride, which could have indirect benefits on kidney function by lowering inflammation and oxidative stress (30, 244, 245). In the heart, glucagon is considered a cardiostimulant that increases cardiac output and heart rate. These effects could alter blood pressure and indirectly alter kidney function to maintain euvolemia (246, 247). Glucagon can increase satiety and reduce food intake and body weight via central nervous system (CNS) and liver-vagus-hypothalamus axis (30). Evidence for a role of GCGR in adipose tissue is disputed with GCGR expression detected in rodent models without concrete evidence for mRNA expression or protein expression in humans (248). Nonetheless, there is evidence that supraphysiological doses of glucagon can stimulate lipolysis in various model species and humans (243). Additionally, if insulin levels are low, glucagon can stimulate thermogenesis in adipose tissue likely mediated by glucagon-stimulated hypoaminoacidemia, fibroblast growth factor 21, and/or CNS-mediated mechanisms (249, 250). Additionally, GCGR activation in the gastrointestinal tract slows gastric emptying (251, 252). Reduction in appetite, food intake, gastrointestinal transit, and increased lipolysis and thermogenesis leads to reduced body weight and adiposity. Reduction in excessive visceral adiposity and body weight can reduce kidney compression and improve electrolyte handling, blood pressure, and microbiota (11). Additionally, GCGR activation in the kidney can directly stimulate thick ascending limb (TAL) electrolyte reabsorption and distal convoluted tubule (DCT) potassium excretion. Collectively, these findings provide evidence that GCGR activation can have both direct and indirect activity on kidney function. In the diagram, solid lines denote direct mechanisms, while dashed lines indicate indirect or secondary mechanisms by which GCGR activation could enhance kidney function. Figure was created with BioRender.com.

Hardman et al. (239, 241) were the first to demonstrate that glucagon administration elevated urinary cAMP levels. Shortly after, the same researchers established that nearly two-thirds of circulating cAMP is freely filtered through the glomerulus, thereby establishing a foundational pathway through which circulating cAMP could exert actions along the nephron (242). The secretion of glucagon-stimulated hepatic cAMP into circulation plays a pivotal role in inhibiting proximal tubule transport and contributes to glucagon-stimulated natriuresis and diuresis (Fig. 3) (198, 253). Notably, direct infusion of cAMP alone results in similar inhibitory effects observed with glucagon administration (198). Additionally, some studies have shown that extrahepatic cAMP can be converted to adenosine all along the nephron, which in turn could activate adenosine receptors and contribute to kidney-related effects of glucagon (254–257).

Within the Loop of Henle, glucagon’s ability to enhance reabsorption is likely, at least partially, mediated by cAMP. Studies indicated that glucagon-stimulated cAMP formation in the kidney occurs predominantly in thick ascending limb and collecting duct (258), a pattern that aligns with more recent RNA sequencing datasets in mice (200, 259) and humans (29, 161, 162). However, the distinct role of glucagon versus cAMP in the Loop of Henle and the collecting duct remains controversial. There is evidence suggesting that cAMP may influence blood pressure through stimulation of renin secretion from juxtaglomerular cells in this region (260). This insight implies that both cAMP and GCG-GCGR actions within the macula densa might propagate downstream signaling that affects tubuloglomerular feedback. However, more studies investigating these topics are necessary. Collectively, this evidence points toward a hepato-renal axis, where glucagon-stimulated hepatic actions, including hepatic-derived cAMP secretion, can exert effects along the nephron that extend beyond the direct actions of glucagon within the kidney.

### GCGR Antagonism versus Agonism

T2D is characterized by elevated blood glucose, which is a consequence of dysregulated circulating levels of insulin and glucagon and insulin resistance (150, 261). Inhibition of glucagon secretion, either directly or indirectly, is considered one of the mechanisms contributing to the favorable outcomes of novel GLP-1-based therapies on glucose control (262–265). Similarly, GCGR antagonists have been shown to improve HbA_1C_ and fasting blood glucose levels in participants with T2D (266–269). However, as discussed earlier, glucagon or GCGR antagonism also show less favorable effects, including increases in liver fat accumulation, circulating cholesterol, liver transaminases, blood pressure, body weight, and stimulation of feeding behavior (268, 270–272). For a detailed review of the topic the reader is directed to Hædersdal et al. (193). These adverse effects limit the clinical usefulness of GCGR antagonists in the treatment of T2D. The influence of GCGR agonism or antagonism on kidney (patho)physiology remains unclear. To our knowledge, the combination of GLP-1RA with GCGR antagonism has not been investigated to determine whether an optimal balance of receptor activity could leverage the desired effects of GLP-1RA and GCGR antagonism, while mitigating the undesired adverse effects of GCGR antagonism.

Nonintuitively, more attention has recently shifted toward GCGR agonism partly due to its role in increasing energy expenditure to combat obesity (273). The addition of GCGRA activity to GLP-1RA is supported by the biological effects of naturally occurring hormone oxyntomodulin (discussed later), which exhibits agonist activity at both GLP-1R and GCGR (274). Emerging preclinical and clinical evidence indicates that the introduction of GCGRA activity into a dual agonist (with GLP-1RA) or triple agonist (with GLP-1RA and GIP receptor agonists) can enhance the management of T2D and obesity (273). The addition of GCGRA activity into incretins can increase fatty acid oxidation, insulin secretion, lipolysis, and energy expenditure and reduce food intake and body weight (30, 273, 275). These combined actions result in robust antidiabetic and antiobesity efficacy for dual and triple incretin analogs with GCGRA activity without the drawbacks observed with GCGR antagonism, if an appropriate balance of receptor activities is maintained (276). While neither GCGR antagonism nor agonism are currently being explored as monotherapeutic options for the treatment of T2D or other metabolic conditions, ongoing efforts are focused on understanding how the beneficial aspects of GCGR agonism can be harnessed in combination with incretin therapies for more efficacious treatment options.

## INCRETIN AND GLUCAGON COMBINATION THERAPIES IN CKD

The pathology of many noncommunicable diseases, including T2D, obesity, and CKD are multifaceted, with multiple dysfunctional pathways contributing to disease progression and comorbidity. It is now evident that combined antidiabetic or antiobesogenic treatments targeting multiple molecular pathways involved in disease progression demonstrate greater efficacy than monotherapies (20, 277, 278). In this section, we delve into possible incretin and glucagon combination therapies and their potential impact on kidney outcomes.

### Incretin and Glucagon Receptor Polypharmacology for CKD

Tirzepatide is the first FDA-approved GLP-1R/GIPR agonist for treating T2D and more recently approved for the treatment of overweight or obesity (16). In patients with T2D, tirzepatide reduced HbA_1c_ below 5.7% in up to 62% of participants at the highest dose (279, 280). In addition, tirzepatide treatment significantly reduced body weight in participants with T2D (281). The magnitude of glycemic control and body weight reduction elicited by tirzepatide is reported to be greater than the leading GLP-1RAs (20, 282). Over half of the patients with overweight or obesity receiving the highest doses of tirzepatide (10 and 15 mg) achieved body weight reductions greater than 20% over 72 wk (16, 283). Although head-to-head studies have not yet been conducted, such a magnitude of body weight reduction has not been observed with GLP-1RA monotherapy (19). A phase 3 clinical study is currently examining the effect of tirzepatide and semaglutide on body weight in participants with obesity (ClinicalTrial.gov identifier NCT05822830). A post hoc analysis of the SURPASS-4 clinical trial investigating tirzepatide versus insulin glargine treatment in participants with T2D showed that tirzepatide slowed eGFR decline over 52 wk, which suggests a kidney-protective effect (21). However, additional studies are necessary to understand tirzepatide’s impact on kidney outcomes. Two active placebo-controlled clinical trials (ClinicalTrial.gov identifier phase 2: NCT05536804 and phase 3: NCT05556512) are investigating tirzepatide’s impact on kidney function (i.e., kidney oxygenation, GFR, and UACR) and composite kidney outcomes (i.e., kidney disease-related death or ESKD, sustained eGFR decline, eGFR slope) as secondary endpoints. Additionally, an active CVOT (ClinicalTrial.gov identifier NCT04255433) will assess tirzepatide’s influence on cardiovascular and kidney outcomes. Nevertheless, dedicated phase 3 studies with kidney outcomes as the primary end point are needed to help establish the kidney benefits of dual GLP-1R/GIPR agonists.

Oxyntomodulin (OXM), a naturally occurring 37-amino acid peptide secreted by the intestine following nutrient intake, is another peptide hormone released from proglucagon’s posttranslational modification with agonist activity at both GLP-1R and GCGR (274, 284–287). The coupling of GLP-1’s anorectic effects with glucagon’s effect on satiety, lipolytic and energy expenditure, offers an appealing rationale for enhanced metabolic improvement (288–298). OXM and its analogs have shown significant metabolic improvements in preclinical models and humans (157, 287, 299–303). However, the potential impact of these compounds on kidney outcomes remains unclear. In preclinical models of diabetic kidney disease, treatment with a dual GLP-1R/GCGR agonist produced significant metabolic improvements (304). In addition, the dual incretin analog improved biomarkers of kidney dysfunction as evidenced by the reductions in plasma creatinine, blood urea nitrogen, and urinary albumin excretion. Cotadutide is an OXM analog with balanced activity at both GLP-1R and GCGR (305). In participants with baseline micro- or macroalbuminuria, cotadutide reduced UACR by 51% after 32 days of treatment, although no differences were observed for eGFR. Cotadutide’s impact on eGFR and kidney outcomes remains to be determined in long-term studies (158). There are several dual agonists under clinical development that target both GLP-1R and GCGR, including survodutide, pemvidutide, efinopegdutide, and mazdutide. While these analogs have been reported to significantly reduce body weight and improve metabolic parameters, their impact on kidney outcomes has not been reported (306–308).

The most recent advance in incretin-based polypharmacology includes the emergence of triple agonists with agonist activity at the GLP-1R/GIPR/GCGR (155, 156, 309). Retatrutide, a novel triple agonist, has demonstrated a mean body weight reduction of 24.2% after 48 wk in participants with obesity who did not have T2D, along with greater reductions in HbA_1c_ compared to GLP-1RA alone in participants with T2D (277, 278). While data from these trials demonstrate improved efficacy in obesity and T2D through the addition of GCGR agonism to GLP-1R/GIPR agonism, whether these synergistic improvements are evident in kidney-related outcomes remains to be determined. An ongoing phase 2 clinical trial (ClinicalTrial.gov identifier NCT05936151) will explore the impact of retatrutide on kidney function in participants with overweight or obesity and CKD with or without T2D. While other triple agonists are in clinical development, cardiovascular or kidney-related outcomes have not yet been disclosed. Although CVOTs are anticipated, studies that specifically investigate kidney-related outcomes are necessary to understand the potential of these therapies in the treatment of CKD.

### Combining Incretin-Based Therapies with Standard-of-Care Treatments in Kidney Disease

SGLT2i effectively treat T2D and CKD by inhibiting the SGLT2 cotransporter in the S1 and S2 segments of the proximal tubule, where ∼97% of total kidney glucose reabsorption occurs, to facilitate glucose and sodium excretion (310, 311). A recent meta-analysis suggested that SGLT2i are superior monotherapies for CKD compared to GLP-1RAs (312, 313). However, combining these therapies may hold the potential for a greater impact on kidney-related outcomes (314). A meta-analysis of 13 studies encompassing 7,350 participants revealed that the combination of GLP-1RA with SGLT2i yields greater reductions in HbA_1c_, body weight loss, and blood pressure compared to SGLT2i alone (315). In patients with T2D, the combination of exenatide and dapagliflozin displayed additive improvements in UACR, surpassing monotherapy and placebo effects (316, 317). Beyond UACR, a post hoc analysis of the EXSCEL CVOT showed a superior reduction in MACE outcomes and eGFR slopes with the combination of exenatide and SGLT2i compared with either monotherapy or placebo (318). The PRECIDENTD trial, a randomized phase 4 clinical trial (ClinicalTrial.gov identifier NCT05390892), aims to directly compare development rates of ESKD events among 9,000 participants with T2D receiving GLP-1RA, SGLT2i, or their combination. Collectively, GLP-1RAs and SGLT2i may offer complementary improvements in kidney outcomes, irrespective of their glucose-lowering or weight-lowering attributes. Further research is warranted to elucidate the mechanisms by which the combination of GLP-1RAs and SGLT2i improves kidney outcomes.

Hypertension commonly coexists with T2D and obesity, with two-thirds of patients with T2D affected (11). Additionally, epidemiological studies estimate that 65–75% of primary hypertension is a consequence of obesity (11, 73). Clinical studies on GLP-1-based therapies show evidence of reduced blood pressure, potentially mediated by RAAS inhibition (16, 17, 20, 277, 319). Preclinical evidence in insulin-resistant rats shows potential benefits in combining ARBs and GLP-1RAs for reducing albuminuria (320). Two case studies also highlight the effective treatment of advanced diabetic nephropathy with a combination of ARB, SLGT2i, and GLP-1RA (321). These studies suggest the potential kidney protective benefits of combining GLP-1RAs with ACEi or ARBs and SGLT2i. However, rigorous investigations are needed to assess safety and efficacy.

## SUMMARY

CKD remains a significant global health concern with limited therapeutic options to halt or slow its progression. In recent years, incretin-based therapies have emerged as novel agents for glucose and body weight reduction in the context of T2D and obesity. Despite the complex pathogenesis of CKD, emerging evidence suggests that GLP-1-based therapies may offer additional benefits in preserving kidney function and improving blood pressure. An understanding of the impact of incretin analogs with the addition of GCGRA activity on kidney function is still at a nascent stage, warranting clinical studies investigating long-term kidney function and outcomes. Further elucidation of molecular mechanisms underlying their role in the kidney and determining their optimal use in combination with standard-of-care treatments could provide promising therapeutic options for patients with CKD.

## DISCLOSURES

All authors are employees or former employees of Eli Lilly and Company.

## AUTHOR CONTRIBUTIONS

B.E.M. prepared figures; B.E.M. and A.K. drafted manuscript; B.E.M., K.L.D., and A.K. edited and revised manuscript; B.E.M., K.L.D., and A.K. approved final version of manuscript.
